# Relationship between Fat Status, Stage of Gonadal Maturity and Hormonal Variation of *Turdus philomelos* (C.L. Brehm, 1831) Wintering in Apulia during 2018–2020

**DOI:** 10.3390/ani14020215

**Published:** 2024-01-09

**Authors:** Simona Tarricone, Antonella Tinelli, Giuseppe Passantino, Nicola Zizzo, Annalisa Rizzo, Antonio Ciro Guaricci, Antonella Perillo, Valeria Buonfrate, Alice Carbonari, Maria Antonietta Colonna, Marco Ragni

**Affiliations:** 1 Department of Soil, Plant and Food Science, University of Bari Aldo Moro, Via Amendola 165/A, 70125 Bari, Italy; simona.tarricone@uniba.it (S.T.); marco.ragni@uniba.it (M.R.); 2 Department of Veterinary Medicine, University of Bari ’Aldo Moro’, S.P. per Casamassima km 3, 70010 Valenzano, Italy; antonella.tinelli@uniba.it (A.T.); giuseppe.passantino@uniba.it (G.P.); nicola.zizzo@uniba.it (N.Z.); annalisa.rizzo@uniba.it (A.R.); antoniociro.guaricci@uniba.it (A.C.G.); valeria.buonfrate@uniba.it (V.B.); 3 Department of Precision and Regenerative Medicine and Ionian Area, University of Bari ‘Aldo Moro’, S.P. per Casamassima km 3, 70010 Valenzano, Italy; antonella.perillo@uniba.it

**Keywords:** *Turdus philomelos*, fat status, gonadal maturity, endocrine status

## Abstract

**Simple Summary:**

The European Birds Directive (2009/147/EC) mandates that migratory game birds “are not hunted during their period of reproduction or during their return to their rearing grounds”. For each huntable species, the study of the onset of the reproductive phase is important in order to plan the hunting season. The purpose of this study was to evaluate the development of the fattening status and reproductive activity of the song thrush. To achieve this goal, the chemical and fatty acid compositions of the pectoral muscle were analyzed in relation to the fattening status of the birds. In addition, reproductive status was evaluated through anatomical and pathological examinations of tissues and the assessment of sex steroid profiles. The results showed that the fattening statuses and fatty acid profiles of the song thrush increase from December to February, corresponding with low ambient temperatures and the approach of spring migration. The change in fatty acid profile may also be related to their diet, which switches from insectivorous to vegetarian, during the winter. The sex hormone profiles and histological examination show that both male and female song thrushes were in a reproductive quiescence phase during the period under consideration.

**Abstract:**

In this study, we aimed to evaluate the development of the fattening condition and the reproductive status of the song thrush from December to February. For this purpose, the chemical and fatty acid compositions of the pectoral muscle were analyzed in relation to the fattening state of the birds. Moreover, their reproductive activity was evaluated via the anatomical and pathological examination of tissues and through the assessment of sex steroid profiles. One hundred ninety-five thrushes captured by local hunters during the 2018–2019 and 2019–2020 hunting seasons in different provinces of the Apulia region in Italy were used. The first step was the measurement of bird body mass, and the amount of subcutaneous body fat was estimated visually. During post-mortem examinations, the pectoral muscle was excised and used for chemical and fatty acid analysis and a hormone assay, respectively. Moreover, ovaries and testicles were evaluated to determine the degree of maturation and thus the reproductive status of the birds. The results regarding fattening status and fatty acid profile confirmed that in January–February, thrushes change their diet, increasing their intake of oleic acid, likely to better cope with low temperatures and prepare for long-distance migration. In both male and female thrushes, the concentrations of sex hormones confirmed a phase of reproductive quiescence from December to February, which was also confirmed through histological examination of the gonads.

## 1. Introduction

Seasonal long-distance migration occurs in all classes of vertebrates [1]. The phenomenon of migration, however, has reached its greatest complexity in birds, which are preadapted to migrate due to their capacity for active flight, size, and extraordinarily efficient circulatory and respiratory systems [2]. Endogenous control mechanisms are thought to regulate seasonally appropriate migratory restlessness and orientation [3] and the onset and end of migratory activity, which may have influences through seasonal changes in dietary and habitat preferences [4] such as the circannual pattern of body mass change, including fat deposition [5,6].

Lipid storage is important for avian reproduction [6], migration [7], and survival [8,9]. Food sources high in unsaturated and high-energy fatty acids may be important for bird migration. After the onset of migration, individuals with larger lipid deposits rich in easily mobilized fats exhibit a greater ability to survive unpredictable and adverse weather conditions and reduced food availability during migration.

In Italy, the song thrush (*Turdus philomelos*) is among the most representative game bird species hunted from December to January. They seem to come to Italy in winter (Italy, Greece, France, Spain, and North Africa), migrating from North-Eastern Europe (Hungary, Poland, Germany, Russia, the Baltic Republics, and the Scandinavian peninsula) [10]. Thrushes arrive in Apulia (Southern Italy) in autumn, between the end of October and the beginning of November, where they stay in closed woods and Mediterranean scrubs, and leave for the breeding grounds between the end of March and the first fortnight of April [11,12]. Variation in the timing of arrival and departure depends on the timing of the breeding season, while the choice of wintering area is related to the environmental conditions and availability of food [13].

The European Birds Directive (2009/147/EC) [14] stipulates that migratory game birds “are not hunted during their period of reproduction or during their return to their rearing grounds”; therefore, for each huntable species, Member States must assess the 10-day period (TDP) in which the pre-nuptial migration starts. For birds wintering in Europe, the onset of northward movement cannot be easily defined. The timing of spring migration is important since the time of arrival at the breeding areas is a significant determinant of reproductive success. The activation of the reproductive state in birds is triggered by exogenous and endogenous stimuli, such as light, temperature, state of nutrition, stress, social interactions, and visual, olfactory, and acoustic signals [15,16].

These stimuli activate the hypothalamic–pituitary–gonadal axis, resulting in the recovery of the reproductive statuses of birds [15,16]. In females, the development of mature ovarian follicles that produce sex hormones, such as estrogens (Estradiol: E_2_), progesterone (P_4_), and testosterone (T), occurs. Estrogens contribute to the development of the follicles themselves, stimulate the liver to begin the synthesis of yolk proteins, and are responsible for reproductive behavior. Furthermore, together with testosterone, they regulate the homeostasis of calcium, which is essential for shell formation [17]. Progesterone, together with testosterone, determines the positive feedback at the hypothalamic level, necessary for inducing ovulation [18]. In males, the testicles produce both testosterone and estrogens during the breeding season. Testosterone is essential for spermatogenesis; the development of secondary sexual characteristics, especially singing; increasing muscle mass; and regulating sexual behavior. Estrogens are required for spermatogenesis and copulatory behavior [17]. 

The aim of the present study was to evaluate the development of the fattening state and the reproductive activity of the song thrush, from December to February, during their wintering in Italy. The chemical and fatty acid compositions of tissue from pectoral muscles and the fat score were analyzed, and the reproductive activity was assessed through the anatomical and pathological examination of tissues and via the assessment of the sex steroid profile. 

## 2. Materials and Methods

### 2.1. Animals

For the survey, 195 thrushes shot in the provinces of Bari, BAT, and Lecce (Apulia region, Italy) were used. The researchers who participated in the experiment were not involved in the hunting of the animals; the local hunters authorized by the Apulian Region freely provided the dead birds for the objectives of this research, without any other purposes.

Specifically, during the years 2018–2020, the following birds were hunted (Table 1):

On 10 February 2019 and 8 February 2020, thrushes were hunted outside the hunting season; thus, they underwent confiscation by the relevant authorities and were released for research purposes.

### 2.2. Analysis

Bird body mass was measured, including feathers, using a 0.01 g precision balance, and birds were plucked in order to evaluate the state of fattening. The amount of subcutaneous body fat of birds was estimated visually and assigned a score from zero (no visible fat on the abdomen or in the furculum) to five (fat clearly visible and bulging on the abdomen and in the furculum), following the Busse and Kania methodology [19].

A post-mortem examination was performed to identify any pathologies and remove the gonads. The pectoral muscle was excised and split into halves, one of which was used for chemical and fatty acid analysis, while the other was used for hormone assays.

#### 2.2.1. Chemical Composition and Fatty Acid Analyses of Pectoral Muscles

To establish the fattening state of each bird, representative sub-samples of the pectoral muscle were pooled and homogenized; AOAC (Association of Official Analytic Chemists, Rockville, MD, USA) procedures were used to assess moisture, ether extract, protein, and ash content [20].

Fat was extracted according to the method suggested by Folch et al. [21], using a 2:1 chloroform/methanol (*v*/*v*) solution to determine the fatty acid profile. The fatty acids were then methylated using a KOH/methanol 2N solution [22] and analyzed via gas chromatography (Shimadzu GC-17A) using a silicone-glass capillary column (70% Cyanopropyl Polysilphenylene-siloxane BPX 70 produced by Thermo Scientific; length = 60 m, internal diameter = 0.25 mm, and film thickness = 0.25 µm). The starting temperature was 135 °C for 7 min; then, it was increased by 4 °C/min up to 210 °C. Samples of each concentrate mixture were used for fatty acid analysis according to the method described above to acquire muscle tissue fatty acid profiles. Fatty acids were expressed as a percentage (wt/wt) of total methylated fatty acids.

#### 2.2.2. Histological and Pathological Examination

All the birds underwent a post-mortem examination in order to determine whether a traumatic impact (i.e., that induced by a firearm) was the cause of death. After evisceration, the abdominal cavity was examined for the identification of the ovaries or testicles. Gonads were fixed in 10% formalin and processed using a Histokinette 2000. The samples were cut to 4 microns using a rotary manual microtome (Leica RM2235, Milan, Italy), mounted on slides and stained with Hematoxylin Eosin (H&E) and Mallory’s trichrome, and subsequently examined under a Leika DM4000 (Leica Microsystems Srl, Buccinasco, MI, Italy) optical microscope at 100× magnification to confirm the sex. In order to observe the developmental state of the gonads in the months between December and February, 5 fields were randomly selected at 400× magnification, identifying the ovary, the testis, and the degree of maturation.

#### 2.2.3. Hormonal Dosages

Steroid hormone concentrations were evaluated following extraction from the pectoral muscles. E_2_, P_4_, and T were evaluated in female thrushes, while E_2_ and T were evaluated in males. In particular, 10 g of tissue was homogenized with 40 mL of phosphate buffer (pH 7.4) in a Stomacher for 1 min. After 45 min of incubation, 1.5 mL of homogenate was poured into an Eppendorf tube and centrifuged to deposit the debris; then, the supernatant was recovered to assess the dosage using the ELISA method. Specifically, the kit was used for T (code 402510; sensitivity: 0.1 pg/mL), E_2_ (code 402110; sensitivity: 20 pg/mL), and P_4_ (code 402410; sensitivity: 0.1 ng/mL). The competitive ELISA test, suitable for hormonal samples extracted from a solid matrix, was performed using a Radim BRIO device.

### 2.3. Statistic Analysis

Compiled forms were entered into an Excel database, and data were analyzed using R software (version 2022.02.0). Data on chemical and fatty acid content are reported as means and standard error of means (SEM). The Shapiro–Wilk test was used to evaluate the normality of the continuous variables, and the Bartlett test was used to evaluate the homostochasticity of the data. Normality of the sample distribution was assessed using the one-way analysis of variance with Duncan’s post hoc test for differences in chemical and fatty acid profiles in different months, and significance was declared at *p* < 0.05. 

One-way ANOVA was used to compare E_2_, T, and P_4_ levels in male thrushes caught in December, January, and February. For all tests, a *p*-value < 0.05 was considered statistically significant.

## 3. Results

### 3.1. Animals

From the macroscopic postmortem examination, the 195 subjects obtained were divided into 72 males and 102 females, while 21 were not suitable. Among the thrushes caught, only 174 had well-preserved gonads, while for the other subjects, the gonad sampling was not suitable due to the traumatic wound inflicted during hunting.

Table 2 shows the results related to thrush body condition. The thrushes (both male and female) hunted in February were significantly (*p* < 0.05) fatter than those caught in December, whereas the body weights were similar in all three months. 

### 3.2. Chemical Composition and Fatty Acid Profile of Thrush Muscle

The chemical composition of the *Pectoralis major* is shown in Table 3. The muscle tissue of the subjects hunted in February showed a significantly (*p* < 0.05) greater concentration of fat as compared to those hunted in December. On the contrary, the thrushes caught during December and January had a higher content of ash (*p* < 0.05) than those hunted in February.

The concentration of Saturated Fatty Acid (SFAs) was significantly (*p* < 0.05) greater in the muscles from thrushes caught in January and February. Among the SFAs, the concentration of palmitic acid (C16:0) was significantly higher in December and January (*p* < 0.01), while the content of stearic acid (C18:0) was markedly higher in January and February (*p* < 0.05). 

The highest concentration (*p* < 0.01) of Monounsaturated Fatty Acids (MUFAs) was recorded in the muscle tissue from thrushes hunted in December and February, as compared to January, and the same pattern was observed for the concentration of oleic acid, which was the most representative monounsaturated fatty acid (*p* < 0.05). 

The content of linoleic acid, DPA, and DHA was significantly higher (*p* < 0.01) in the muscles of thrushes caught in January and February than those caught in December; these results led to a significantly (*p* < 0.01) greater value of total Polyunsaturated Fatty Acids (PUFAs).

Consequently, the concentration of total Unsaturated Fatty Acids (UFAs) was also significantly greater in January and February compared to that in December (*p* < 0.01).

### 3.3. Histological Examination

**Figure 1:**
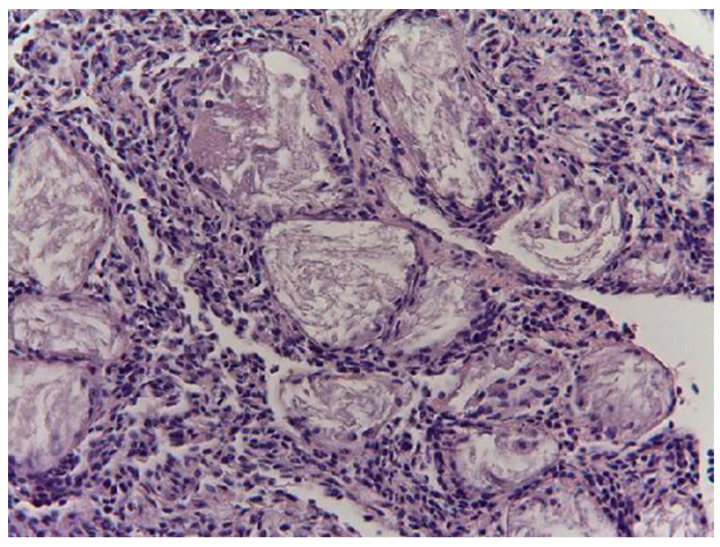
Primordial and primary oocytes in a female song thrush hunted in December (H&E, 200×).

Primordial and primary oocytes were present in the ovarian cortex of the females hunted in December (Figure 1). The most represented primordial oocytes were organized in clusters, surrounded by a row of flat cells with a spherical or eccentric nucleus with an evident nucleolus and filamentous-looking ooplasm, delimited by a layer made of collagen fibers, elastic fibers, and fibroblasts. A shrunken medulla occupied the central part of the ovary and had slightly ectatic blood vessels.

**Figure 2:**
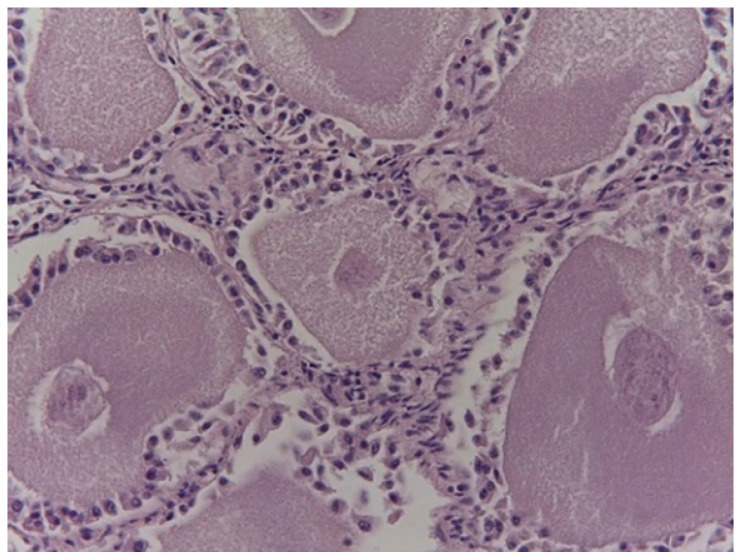
Oocytes with acidophilic and homogeneous ooplasm and central nucleus from birds hunted in late January.

The primary oocytes showed a greater volume than the primordial ones. They were surrounded by a layer of cubic cells with a spherical and vesicular nucleus with an evident nucleolus. The ooplasm was acidophilic, homogeneous, and compact, showing rounded structures, which were granules of albuminoid substance (Figure 2). When visible, the nucleus was located in a central or eccentric position. The primary oocyte was externally enveloped by a layer of cells that was thicker than that observed in primordial oocytes. The primordial and primary oocytes of different sizes occupying the cortex caused it to expand further, reducing the space of the medulla until the clear separation between the two regions disappeared.

**Figure 3:**
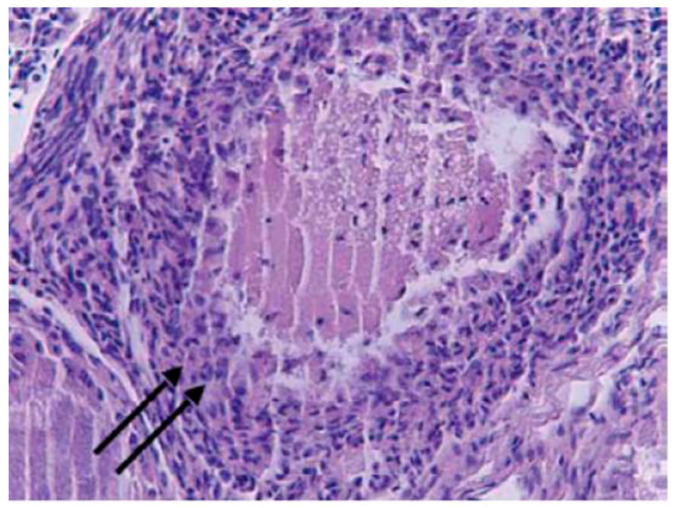
Secondary oocyte with follicular theca in a female song thrush hunted in February (H&E, 400×).

The birds caught in January presented the same morphological characteristics, while those hunted in February showed secondary oocytes, characterized by cytoplasm with a more colored central region with paler fine granules and peripheral vacuoles. The nucleus assumed, in this stage, an eccentric position. The perivitelline membrane, which was very acidophilic and surrounded by numerous cubic cells of stratum granulosum, showed more than one layer. The theca of the secondary follicle, on the other hand, was composed of two distinct layers: the internal theca, which was denser and presented parallel collagen fibers, abundant reticular fibers, and scarce elastic fibers, and the external theca, which was thicker and characterized by collagen fibers arranged in a looser manner, reticular fibers, and slight elasticity; at this stage, the distinction between the cortex and the medulla disappeared (Figure 3).

**Figure 4:**
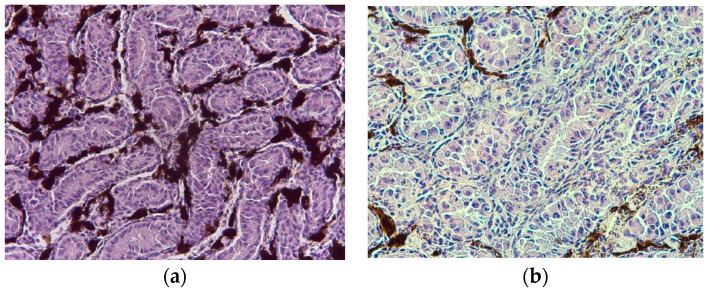
(**a**) Testicles: seminiferous tubules with primary and secondary spermatocytes (H&E, 100×); (**b**) Details of the seminiferous tubules with the germ line in different maturation stages (H&E, 100×).

In the testicles of the males hunted in December, seminiferous tubules showed walls containing two to three layers of cells (Figure 4), characterized mostly by spermatogonia, followed by primary and secondary spermatocytes. In January and February, spermatids and some spermatozoa were present.

Among these cytotypes representing the stages of spermatogenesis and spermiogenesis, there were also Sertoli cells, located in the connective tissue surrounding the seminiferous tubules of the testicles, which had the function of supporting the germ cells during the subsequent maturation stages. Over the three months analyzed, the Sertoli cells gradually increased in number and volume. 

### 3.4. Hormonal Profile

The results regarding the concentrations of male and female reproductive hormones are shown in Table 4.

The concentrations of E_2_ in female song thrushes increased from December to February, although not significantly, while the concentrations of P_4_ and T remained similar over the sampling periods.

In male thrushes, no significant differences in terms of T and E_2_ levels were observed from December to February. 

## 4. Discussion

The results presented in this study provide, for the first time, knowledge about the chemical and fatty acid compositions of the muscle tissue and the concentration of reproductive hormones in song thrushes caught in Apulia during the 2018/2019 and 2019/2020 hunting seasons.

The state of fattening revealed increasing deposits of cover fat, which reached a maximum in February, in correspondence with the lowest ambient temperatures and the beginning of the spring migration in March. The chemical composition of the pectoral muscle tissue highlighted that the intramuscular content of fat was highest in February; this result may be related to the thrushes’ diet, which changes from an insectivorous to vegetarian feeding regimen during the wintering period. This feeding is characterized by the consumption of nutritious oleaginous fruits such as olives, which help to satisfy thrushes’ need to increase their food ingestion and fat deposits to enable high-intensity exercise and flight, close to the migration period.

The fatty acid profile of the pectoral muscle changes in relation to environmental conditions [23,24,25] and food ingested by animals [26,27]; in particular, muscle tissue from thrushes caught during January and February showed the highest concentration of SFAs, which may be useful for the deposition of cover fat contributing to the regulation of body temperature. The concentration of oleic acid (C18:1), the main fatty acid present in olives, was greatest in muscle tissue from thrushes hunted in February, thus leading to the higher concentration of total MUFAs and, accordingly, supporting the findings reported by other authors regarding birds hunted in winter [28,29]. Also, Rodríguez-Turienzo et al. [30] found higher levels of oleic acid in muscle tissue from thrushes shot in Spain during the winter. The energy content of fatty acids increases with chain length and decreases with unsaturation; C18-chain fatty acids provide relatively great energy content per unit mass [31]. Indeed, migratory birds, which need to store large amounts of fat, store more C18:1 compared to non-migrants [32]. Therefore, adipose tissues can reach colder temperatures without solidifying. The ability to cool their superficial fat tissues helps an animal to create a thermal gradient, which contributes to body heat loss reduction. Therefore, as found in mammals [33], birds exposed to colder environments show adipose tissues with higher proportions of unsaturated fat than birds living in temperate climate conditions. 

Accumulation of easily mobilized, long-chain, low-melting-point, unsaturated fatty acids may be an adaptive response for thrushes prior to a long-distance migration wherein availability and distribution of food resources are unpredictable. This hypothesis is confirmed by our results, which show an initial accumulation of polyunsaturated fatty acids in January and February necessary for migration. This finding also has potential implications for human health since MUFAs and PUFAs have well-documented effects on preventing cardiovascular dysfunctions [34,35].

As for the hormonal assays, in female song thrushes, only the estradiol level showed a slight increase from December to February. In avian species, estrogens are involved in follicular development but do not contribute, unlike in mammals, to the development of the LH surge, which is necessary for triggering ovulation [17]. Therefore, it is conceivable that this increase could be due to the presence of small follicles that are not yet preovulatory [17]. In confirmation of the state of reproductive quiescence of this period, the concentrations of progesterone and testosterone, which are essential for triggering ovulation [17], were almost the same in December and February. The absence of ovulation was also identifiable via morphological alterations characterized by a reduction in the volume of the ovum, the disorganization of the case, the permanence of interstitial cells, and the invasion of the lumen by cells of the granulosum layer.

In male thrushes, no significant changes in the hormonal profile were detected in February either, which is closer to the onset of the April–May breeding period than December. In general, we observed a decrease in the estradiol concentration from December to February, while the testosterone level showed a slight increase. This result corresponds with those obtained for the females, confirming the state of reproductive rest in this period. The histological examinations highlighted the state of rest of both sexes’ gonads, while male thrushes showed a slight increase in spermatogenesis and a low presence of sperm towards February.

## 5. Conclusions

During December–February, in both female and male song thrushes, the concentration of sex hormones did not indicate functional gonads, thus confirming the phase of reproductive quiescence. These results were further confirmed through histological investigations, where the morphological characteristics showed incomplete maturation of the ova and spermatozoa. 

The increase in intramuscular fat during February suggests the beginning of the fat deposition process that prepares birds for the onset of migration. Further investigations are needed to study this species in late February and March as well in order to increase knowledge about the onset of the migration process and changes in tissue distribution, such as the increase in fat deposits, with respect to the song thrush. These results will provide important information to the local authorities, enabling them to more accurately schedule the hunting seasons in Apulia.

## Figures and Tables

**Table 1:** Birds hunted during the 2018/2019 and 2019/2020 hunting seasons.

	2018/2019	2019/2020
December	40	40
January	40	40
February	20	15
Total	100	95

**Table 2:** Thrush body condition.

	December	January	February	SEM ^1^	*p*-Value
Body mass (g)	67.07	67.25	67.28	0.044	0.074
Body weight without feathers (g)	60.85	60.51	60.66	0.054	0.985
State of fattening in males	2.40 ^b^	3.00 ^ab^	4.00 ^a^	0.042	0.048
State of fattening in females	2.51 ^b^	3.00 ^ab^	4.00 ^a^	0.202	0.049

^1^ SEM: Standard error of means; ^a, b^: *p* < 0.05.

**Table 3:** Chemical composition and fatty acid profiles of thrush pectoral muscle (%).

Title 1	December	January	February	SEM ^1^	*p*-Value
Moisture	70.04	70.00	70.11	0.638	0.084
Protein	21.07	20.87	21.25	0.652	0.070
Ether extract	4.75 ^b^	4.99 ^ab^	5.20 ^a^	0.838	0.045
Ash	4.14 ^a^	4.14 ^a^	3.10 ^b^	0.067	0.039
Fatty acid profile (% FAME) ^2^					
C14:0	0.12	0.08	0.05	0.115	0.074
C16:0	11.54 ^A^	11.43 ^A^	10.74 ^B^	0.758	0.005
C17:0	0.11	0.11	0.12	0.115	0.074
C18:0	8.84 ^b^	10.50 ^a^	10.81 ^a^	1.192	0.043
C20:0	0.17	0.25	0.25	0.613	0.535
C22:0	0.02	0.03	0.03	0.003	0.213
∑ SFA	20.80 ^b^	22.40 ^a^	22.00 ^a^	2.050	0.035
C14:1	0.08	0.04	0.09	0.005	0.422
C16:1 *trans*	0.26	0.24	0.31	0.220	0.256
C16:1 *cis*	0.99	0.69	0.87	0.229	0.218
C18:1 n−9 *cis* (oleic acid)	61.05 ^a^	55.98 ^b^	64.60 ^a^	2.461	0.047
∑ MUFA	62.38 ^A^	56.95 ^B^	65.69 ^A^	2.801	0.002
C18:2 n−6 (linoleic acid)	8.14 ^B^	9.52 ^A^	9.06 ^A^	1.124	0.005
C18:3 n−6 (γ−linolenic acid)	0.33	0.27	0.29	0.238	0.165
C18:3 n−4	0.15	0.16	0.13	0.073	0.316
C18:3 n−3 (α-linolenic acid)	0.23	0.24	0.23	0.186	0.240
C18:4 n−3	0.07	0.04	0.07	0.047	0.060
C20:2 n−6	0.04	0.04	0.03	0.086	0.071
C20:3 n−6	0.08	0.09	0.11	0.116	0.229
C20:4 n−6 (ARA)	2.21	2.94	2.45	0.587	0.237
C20:4 n−3	0.06	0.11	0.09	0.047	0.060
C20:5 n−3 (EPA)	0.11	0.13	0.13	0.080	0.332
C22:5 n−6	0.13	0.14	0.13	0.073	0.242
C22:5 n−3 (DPA)	0.47 ^B^	0.57 ^A^	0.53 ^A^	0.086	0.001
C22:6 n−3 (DHA)	4.41 ^B^	5.89 ^A^	6.19 ^A^	0.714	0.007
∑ PUFA	16.43 ^B^	20.14 ^A^	19.44 ^A^	1.431	0.007
∑ UFA	78.81 ^B^	77.09 ^B^	85.13 ^A^	0.798	0.006

^1^ SEM = standard error of means; ^2^ FAME—total fatty acid methyl esters; SFAs—saturated fatty acids (sum of C14:0 + C16:0 + C17:0 + C18:0 + C20:0 + C22:0); MUFAs—monounsaturated fatty acids (sum of C14:1 + C16:1 *trans* + C16:1 *cis* + C18:1 n−9 *cis*); PUFAs—polyunsaturated fatty acids (sum of C18:2 n−6 + C18:3 n−6 + C18:3 n−4 + C18:3 n−3 + C18:4 n−3 + C20:2 n−6 + C20:3 n−6 + C20:4 n−6 + C20:4 n−3 + C20:5 n−3 + C22:5 n−6 + C22:5 n−3 + C22:6 n−3); UFAs—unsaturated fatty acids (sum of MUFAs + PUFAs); ^a, b^: *p* < 0.05; ^A, B^: *p* < 0.01.

**Table 4:** Male and female reproductive hormonal profiles (mean ± SD).

	Male		Female		
	E_2_ * (pg/mL)	T * (pg/mL)	E_2_ (pg/mL)	P_4_ * (ng/mL)	T (pg/mL)
December	60.53 ± 15.47	1.15 ± 0.33	46.52 ± 19.88	0.49 ± 0.24	1.14 ± 0.40
January	55.89 ± 21.38	1.21 ± 0.38	63.99 ± 25.14	0.44 ± 0.21	1.12 ± 0.36
February	57.68 ± 21.74	1.17 ± 0.26	63.18 ± 22.94	0.36 ± 0.18	1.03 ± 0.23
*p*-value	0.8311	0.9248	0.1151	0.6662	0.9314

* E_2_: Estradiol; T: Testosterone; P_4_: Progesterone.

## Data Availability

Data are contained within the article.
